# Predicting interindividual response to theta burst stimulation in the lower limb motor cortex using machine learning

**DOI:** 10.3389/fnins.2024.1363860

**Published:** 2024-03-20

**Authors:** Natsuki Katagiri, Tatsunori Saho, Shuhei Shibukawa, Shigeo Tanabe, Tomofumi Yamaguchi

**Affiliations:** ^1^Department of Rehabilitation Medicine, Juntendo University Graduate School of Medicine, Tokyo, Japan; ^2^Department of Rehabilitation Medicine, Tokyo Bay Rehabilitation Hospital, Chiba, Japan; ^3^Department of Radiological Technology, Kokura Memorial Hospital, Fukuoka, Japan; ^4^Department of Radiological Technology, Faculty of Health Science, Juntendo University, Tokyo, Japan; ^5^Center for Evolutionary Cognitive Sciences, Graduate School of Art and Sciences, University of Tokyo, Tokyo, Japan; ^6^Department of Radiology, Tokyo Medical University, Tokyo, Japan; ^7^Faculty of Rehabilitation, School of Health Sciences, Fujita Health University, Aichi, Japan; ^8^Department of Physical Therapy, Faculty of Health Science, Juntendo University, Tokyo, Japan; ^9^Department of Physical Therapy, Yamagata Prefectural University of Health Sciences, Yamagata, Japan

**Keywords:** noninvasive brain stimulation, cortical plasticity, machine learning, interindividual variability, primary motor cortex, lower limb, transcranial magnetic stimulation

## Abstract

Using theta burst stimulation (TBS) to induce neural plasticity has played an important role in improving the treatment of neurological disorders. However, the variability of TBS-induced synaptic plasticity in the primary motor cortex prevents its clinical application. Thus, factors associated with this variability should be explored to enable the creation of a predictive model. Statistical approaches, such as regression analysis, have been used to predict the effects of TBS. Machine learning may potentially uncover previously unexplored predictive factors due to its increased capacity for capturing nonlinear changes. In this study, we used our prior dataset (Katagiri et al., 2020) to determine the factors that predict variability in TBS-induced synaptic plasticity in the lower limb motor cortex for both intermittent (iTBS) and continuous (cTBS) TBS using machine learning. Validation of the created model showed an area under the curve (AUC) of 0.85 and 0.69 and positive predictive values of 77.7 and 70.0% for iTBS and cTBS, respectively; the negative predictive value was 75.5% for both patterns. Additionally, the accuracy was 0.76 and 0.72, precision was 0.82 and 0.67, recall was 0.82 and 0.67, and F1 scores were 0.82 and 0.67 for iTBS and cTBS, respectively. The most important predictor of iTBS was the motor evoked potential amplitude, whereas it was the intracortical facilitation for cTBS. Our results provide additional insights into the prediction of the effects of TBS variability according to baseline neurophysiological factors.

## 1 Introduction

Theta burst stimulation (TBS) modulates cortical excitability and induces cortical plasticity; these enhance motor functional recovery in patients with neurological disorders (Huang et al., 2005; Somaa et al., 2022); however, inter-and intra-individual variability of TBS-induced plasticity prevents its clinical application (Terranova et al., 2018). Hence, to create a predictive model that may distinguish between responders and nonresponders to TBS according to the baseline neurophysiological status, it is necessary to examine the factors associated with this variability.

Previous meta-analysis has shown that individual differences in TBS effects on the upper limb motor cortex may be predicted based on the baseline amplitude of motor evoked potentials (MEP) elicited by transcranial magnetic stimulation (TMS) (Corp et al., 2020). However, an effective model that can predict TBS-induced plasticity in the lower limb motor cortex has not yet been established. Notably, the lower limb representation within the motor cortex is positioned deeper than that of the hand. Moreover, the motor cortex layer is parallel to the sagittal plane, and the bilateral regions are close to each other (Huang et al., 2018). Hence, the factors that may predict the effects of TBS may also differ as the induction of electric fields by TBS varies between the leg and hand regions.

Despite our previous results, no significant neurophysiological factors that effectively classify responders and nonresponders to TBS of the lower limb motor cortex have been identified (Katagiri et al., 2020). One difficulty contributing to this result may be attributed to the nonlinear interindividual variability observed in TBS-induced plasticity. Previous meta-analyses have demonstrated a nonlinear negative relationship between changes in MEP following TBS and amplitudes of MEP at baseline measured to 120% of the resting motor threshold (RMT) or 1 mV, which represents the MEP amplitude on the rising phase of the stimulus–response curve wherein a roughly linear increase with TMS intensity before intervention can be observed (Rossini et al., 2015; Corp et al., 2020). Using linear or logistic regression analysis, which is designed for capturing linear changes, prior studies have attempted to predict the effects of TBS (López-Alonso et al., 2014; Corp et al., 2020; Katagiri et al., 2020). However, this analysis is limited in cases wherein nonlinear relationships must be addressed as it attempts to fit a linear model to the dataset (Ray, 2019). Therefore, employing analytical techniques that can capture nonlinear variations is essential for developing models that may accurately distinguish TBS responders from nonresponders.

Supervised machine-learning methods for investigating large and complex datasets are novel approaches in natural science (Jordan and Mitchell, 2015; Vu et al., 2018; Albizu et al., 2020; Tozlu et al., 2020; Wessel et al., 2021). Machine learning can capture nonlinear changes, allowing for the prediction of the effects of noninvasive brain stimulation (NIBS) from data that cannot be captured by regression analysis, in contrast to linear relationships.

Employing nonlinear analysis through machine learning is a promising option for secondary analysis of our existing dataset (Katagiri et al., 2020). In the literature, associations between the effects of NIBS and neurophysiological parameters, including short-interval intracortical inhibition (SICI), intracortical facilitation (ICF), and the coefficient of variation of the MEP (MEP-CV), have been reported (Hordacre et al., 2017; Li et al., 2019; Katagiri et al., 2020). Additionally, the slope of the recruitment curve (RC) is a predictor of variability in the amplitude of the test MEP (Sarkar et al., 2022). By incorporating these factors, machine-learning techniques may effectively identify responders and nonresponders, subsequently complementing the traditional linear regression approach used in previous studies. In this study, the primary objective was to employ machine learning to our prior dataset for secondary analysis and to reveal the determining factors at baseline for each TBS response. Machine learning was used to create an optimized ensemble model for predicting TBS responses in the lower limb motor cortex.

## 2 Materials and methods

### 2.1 Subjects

This study enrolled 48 healthy participants who were recruited for a previous study (Katagiri et al., 2020). The full details of the procedure, TBS setting, and electromyography results were reported in a previous study (Katagiri et al., 2020). Responders and nonresponders were derived by two-step cluster analysis of the mean changes in MEP immediately after each TBS (Katagiri et al., 2020). We used the changes of MEP as the criterion for defining responders based on reports indicating that TBS application to M1 modulates the motor cortex, with MEP serving as the primary outcome measure of corticospinal excitability (Huang et al., 2005). All participants provided written informed consent before participation. This study was approved by the ethics committee of the Faculty of Health Science, Juntendo University (approval number: 20-020), and was performed in accordance with the Declaration of Helsinki.

### 2.2 Data preprocessing and machine-learning modeling

To resolve the class imbalance within the dataset, synthetic minority oversampling technique (SMOTE) with a k-neighbor parameter set to 5 was employed. Additionally, the Pycaret library (version 3.1.0) was used to improve the procedural efficiency of our methodology. This library streamlined the implementation of diverse machine-learning models, resulting in a systematic and expedited workflow.

During the machine-learning modeling phase, the training dataset was meticulously configured by allocating 70% of the data for training (train_size). Using the normalized parameter, feature standardization was performed. Elimination of highly collinear features depended on binary determination based on a specific threshold. Additionally, preservation of label equilibrium during data splitting was ensured.

Our methodology incorporated k-fold cross-validation, with the parameter fold indicating the number of folds employed. This methodological care was created to refine the model for optimal accuracy. The integration of Pycaret not only facilitated seamless comparisons among multiple machine-learning models but also enabled the construction of an optimized ensemble model, with classifiers encompassing decision trees, random forest, gradient boosting, support vector machine, and k-nearest Neighbors.

The performance of the model was evaluated using the area under the curve (AUC), confusion matrix, and determination of feature importance, providing insights into the model’s efficacy and robustness.

Incorporating Pycaret into our procedural framework streamlined the machine-learning pipeline, ensuring efficiency without compromising the stringency of model evaluation. This combination of sophisticated techniques and tools remarkably enriched the depth and breadth of our analytical approach (Moharekar et al., 2022; Katsuki et al., 2023).

To evaluate the performance of the learning models, the following metrics were obtained in this study. A score approaching 1 indicated a higher performance.

AUC: The receiver operating characteristic (ROC) curve shows the trade-off between the model’s true positive and false positive rates. AUC represents the area under the ROC curve, with a higher AUC value suggesting superior model performance.

Accuracy: Accuracy reflects the proportion of correctly classified instances among all predictions and is calculated as (True Positives + True Negatives)/Total Data.

Precision: Precision indicates the proportion of instances predicted as positive that were indeed positive and is calculated as True Positives/ (True Positives + False Positives).

Recall: Recall represents the proportion of actual positive samples correctly identified as positive by the model as calculated as True Positives/ (True Positives + False Negatives).

F1 Score: The F1 Score is the harmonic mean of precision and recall and is calculated as 2 (Precision * Recall)/(Precision + Recall).

The results of these metrics will contribute to a comprehensive understanding of the learning models’ diagnostic efficacy. For this study, the learning model with the highest performance across all metrics was adopted.

To predict changes in motor cortex excitability after TBS, we assessed SICI, ICF, slope of RC, and MEP-CV. As inhibitory or facilitatory interneurons play an important role in TBS-induced plasticity of the cortical excitability of stimulated regions (Li et al., 2019), we hypothesized that excitability by intracortical inhibition or facilitation before TBS may predict the interindividual variability of TBS-induced corticospinal excitability. We then applied a subthreshold conditioning paired-pulse paradigm to test SICI and ICF (Kujirai et al., 1993). The interstimulus intervals (ISIs) were set at 2.5 ms (SICI) and 10 ms (ICF), with 15 trials being recorded for each ISI and test stimulation. An ISI of 2.5 ms was selected to avoid mixing different SICI mechanisms (Fisher et al., 2002).

To explore the predictive factors of response to TBS, an RC was generated. TMS intensities increased by 20% per step, from 80 to 200% of the active motor threshold. According to the data points, regression plots were fit to the approximately linear part of the RC, and the slope of the RC, which reflects the gain in MEP amplitude with increasing stimulus intensity, was then calculated (Hardwick et al., 2014). Proton magnetic resonance spectroscopy showed a positive correlation between slope and cortical glutamate levels in the motor cortex, suggesting an association between glutamatergic neurotransmission and corticospinal excitability (Stagg et al., 2011; Rossini et al., 2015).

Additionally, we calculated the MEP-CV as follows: MEP-CV = standard deviation (SD)/mean peak-to-peak MEP amplitude. MEP-CV is reportedly associated with the response to cTBS in the hand motor cortex area (Hordacre et al., 2017). Hence, for all TMS measurements, 15 stimuli were delivered every 5 s at each time point in pseudorandom timing. Raw waveforms wherein muscle contractions over 10 μV were mixed were rejected and remeasured. Considering the amplitude variability, the first waveform was removed from all TMS tests. Then, waveforms that exceeded ±2 SD, as calculated from the amplitudes of 14 waveforms, were removed.

Furthermore, International Physical Activity Questionnaire (iPAQ) was administered to each participant and incorporated into the analysis to evaluate physical activity factors. This decision was informed by prior research indicating that the routine physical activity level influences the efficacy of NIBS (Cirillo et al., 2009).

## 3 Results

### 3.1 Interindividual variability in TBS response

Based on our research (Katagiri et al., 2020), two-step cluster analysis identified two clusters for both iTBS and cTBS. One cluster aligned with findings from a prior investigation on the effects of each TBS on the upper limb primary motor cortex (Huang et al., 2005), whereas the other exhibited either no effects or had effects in the opposite direction. We categorized the cluster demonstrating motor cortex excitability modulation similar to that in previous studies as “responders,” whereas the cluster that exhibited inconsistent modulation was the “nonresponders” (López-Alonso et al., 2014).

Regarding our previous report (Katagiri et al., 2020), 27% of participants (13/48) showed a significant increase in MEP amplitude at Post-0 of the iTBS protocol, while 63% (30/48) showed a significant decrease in MEP amplitude after the cTBS protocol. Furthermore, 73% of the participants (35/48) were classified as nonresponders to iTBS, while 38% (18/48) were nonresponders to cTBS. Moreover, 21% of the participants (10/48) were identified as responders to both TBS protocols. The mean percentage of baseline MEP (SD) for each cluster immediately after TBS was 125.5% (16.4%) for responders to iTBS, 104.1% (27.4%) for nonresponders to iTBS, 79.4% (13.5%) for responders to cTBS, and 106.4% (15.2%) for nonresponders to cTBS. The normalized MEP amplitudes in iTBS responders were significantly higher than those in iTBS nonresponders at Post-0 (*p* < 0.001). Additionally, the normalized MEP amplitudes in cTBS responders were significantly lower than those in cTBS nonresponders at Post-0 (*p* < 0.001).

### 3.2 Predictions of interindividual variability in the TBS response

The learning models demonstrated excellent performance on the iTBS and cTBS datasets. In both models, the Extra Tree model emerged as the best learning model. For the Extra Tree model, optimization was performed by maximizing AUC, and the evaluation metrics at the optimized points are presented in Table 1. Notably, iTBS exhibited superior performance.

**Table 1:** The table depicits the Accuracy, Precision, Recall, and F1 scores for each TBS’s Extra Trees model.

	Accuracy	Precision	Recall	F1
iTBS	0.762	0.818	0.818	0.818
cTBS	0.722	0.667	0.667	0.667

The comprehensive metrics showcase superior results for the iTBS.

**Figure 1:**
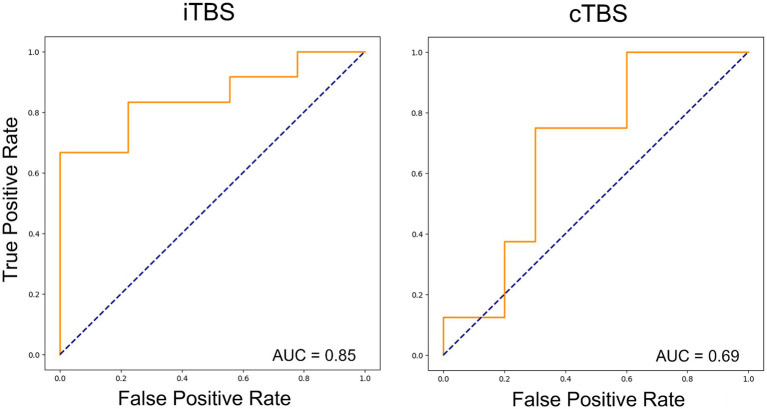
Receiver operating characteristic curves for the two types of theta burst stimulation (TBS) using machine-learning models. In both cases, the Extra Tree model showed the best performance, and learning optimization was performed using the area under the curve (AUC) as the optimization metric. The results showed an AUC of 0.85 and 0.69 for iTBS and cTBS, respectively.

The results emphasize that Extra Tree models were optimized based on AUC considerations. The ROC curves and corresponding AUC values for the performance of the Extra Tree model on each dataset are shown in Figure 1. With the AUC as the optimization metric, the resulting AUCs were 0.69 for cTBS and 0.85 for iTBS.

**Figure 2:**
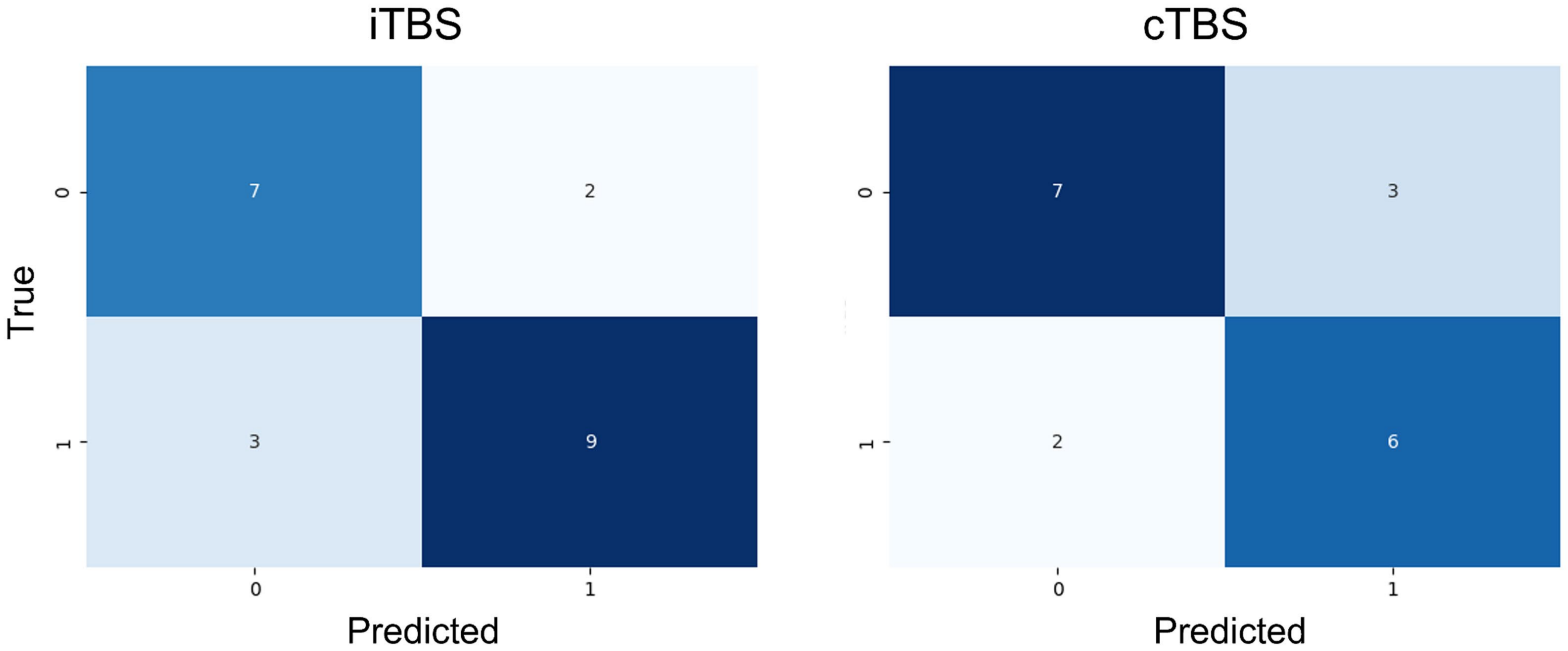
Confusion matrix for the employed machine-learning model (Extra Trees) on the test data. Both models demonstrated relatively accurate classification of unknown data.

The confusion matrices that show the response of the test data for the two Extra Tree models are shown in Figure 2. The true positive rates were 77.7 and 70.0%, whereas the true negative rates were both 75.5% when applied to unknown data not used for training.

**Figure 3:**
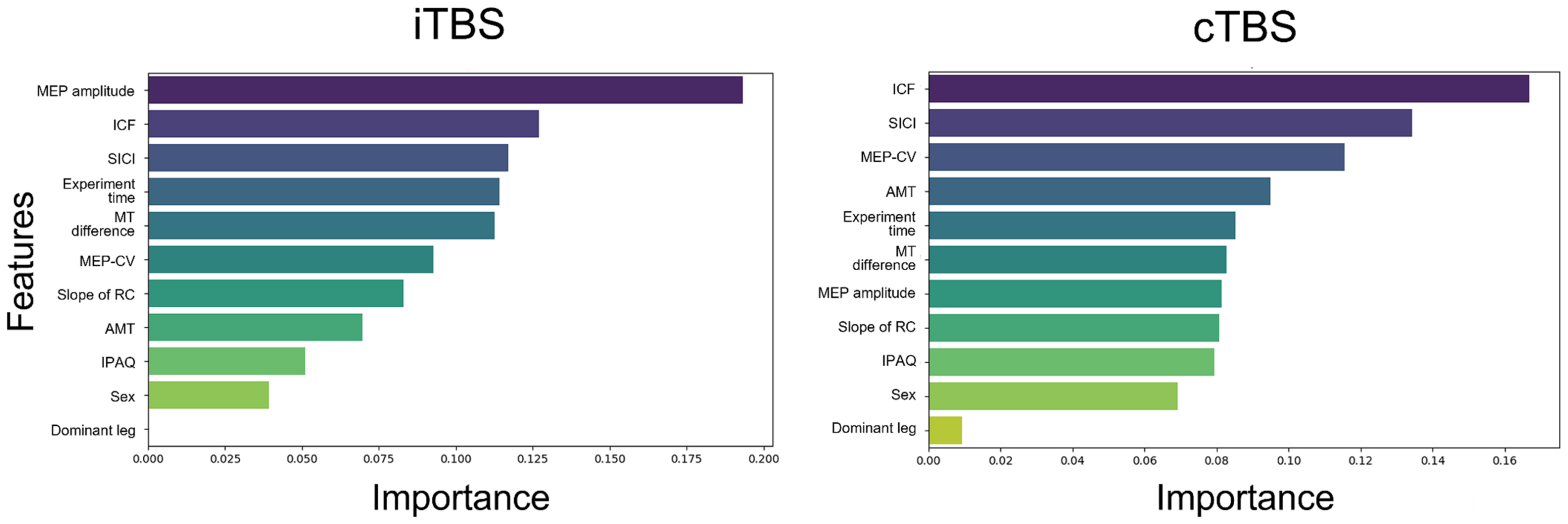
The importance of features in the learning of each model was assessed. The following features were used in the analysis: MEP amplitude, intracortical facilitation (ICF), short-interval intracortical inhibition (SICI), experiment time, the difference between resting and active motor thresholds (MT difference), coefficient of variation of MEP (MEP-CV), slope of the recruitment curve (RC), active motor threshold (AMT), International Physical Activity Questionnaire (IPAQ) scores, sex, and dominant leg. For iTBS, the importance of features associated with corticospinal excitability was emphasized. For cTBS, features related to intracortical excitability, including ICF and SICI, were highlighted as important.

The importance of each feature in label classification is shown in Figure 3. For cTBS, the most important feature, as indicated by ICF, was the excitability of intracortical neurons mediating the primary motor cortex. Conversely, for iTBS, the most important feature was the MEP amplitude, which was an index of excitability in the corticospinal pathway. In both learning models, variables, such as activity levels from the iPAQ, and information from categorical variables, such as gender or foot dominance, provided limited insights.

## 4 Discussion

To the best of our knowledge, this study is the first to predict the effects of TBS on cortical excitability in the lower limb motor cortex using a machine-learning model. Our results show that the effects of iTBS on the lower limb motor cortex are based on the MEP amplitude and degree of intracortical excitability, whereas those of cTBS are based on the degree of ICF and SICI. These findings suggest the potential use of machine learning to assist decision making regarding the application of TBS to the lower limb motor cortex in patients with neurological disorders.

### 4.1 Differences in the prediction of machine-learning models and statistical analysis

Logistic regression analysis has been used to predict the variability in the TBS effects on the lower limb motor cortex (Katagiri et al., 2020), but no significant factors were identified. In contrast, this study employed machine learning using the Extra Trees model, allowing for the exploration of multiple predictors. Furthermore, by using a small dataset that was not used for training, our machine-learning algorithm allowed Extra Trees to identify TBS responders and nonresponders with >70% accuracy according to the baseline neurophysiological characteristics. To the best of our knowledge, no studies have also used machine-learning methods incorporating baseline neurophysiological factors as features to predict the variability of MEP changes following TBS.

Logistic regression relies on the assumption of linearity in the logit for continuous variables (Stoltzfus, 2011). Meanwhile, machine-learning methods, including Extra Trees, effectively capture nonlinear relationships and interactions (Nusinovici et al., 2020). The results of this study show that by comprehensively capturing nonlinear neurophysiological relationships, Extra Trees can identify influences that logistic regression analyses may miss. This also suggests that machine learning could help interpret neurophysiological data, revealing factors of TBS effects that traditional statistical methods may not fully capture. The exploration of machine learning for predicting MEP variability after NIBS is a novel approach in this field of study.

### 4.2 Differences in predictors of variations in TBS patterns

In the iTBS condition, the MEP amplitude was identified as a crucial predictive factor, and the AUC of the model was good. In previous studies, MEP amplitude has been identified as an iTBS predictive factor, indicating that more significant effects would be associated with smaller amplitudes (Corp et al., 2020; Leodori et al., 2021). Small amplitudes might imply facilitatory synaptic activity reduction on pyramidal neurons. In addition, Leodori and colleagues reported that beta oscillations, assessed using electroencephalography (EEG) are predictive factors also (Leodori et al., 2021). According to the metaplasticity theory, the plasticity of a neuron depends on its initial functional state (Suppa et al., 2016), indicating that neuronal oscillatory activity could influence individual responses to TMS. The prestimulus beta neural oscillations reportedly modulated test TMS-induced MEP amplitudes (Mäki and Ilmoniemi, 2010; Hussain et al., 2019). These findings suggest that the initial functional state of motor cortical facilitatory synaptic activity on the motor cortex might impact the subsequent iTBS effects.

Conversely, ICF was identified as a contributing factor in cTBS. However, it is essential to note that the AUC of the model was poor. The low AUC value could be attributed to the unclear origin of ICF and the insufficiency of predictive factors during creation of cTBS model. ICF is thought to reflect the activation of glutamate mediated *N*-methyl-d-aspartate excitatory interneurons in the motor cortex, though the detailed mechanisms remain elusive (Rossini et al., 2015). It is indicated that ICF conditioning TMS specifically facilitates the P60 of TMS-evoked EEG potentials (Cash et al., 2017). The 60 components are linked to glutamatergic neurotransmission, mainly localized in the primary somatosensory cortex (Ahn and Fröhlich, 2021; Belardinelli et al., 2021). These findings imply that the state of pre-existing glutamatergic neural components within the sensorimotor cortex might be crucial for subsequent cTBS effects.

### 4.3 Differences in predictors of TBS to the upper and lower limb motor cortex

Similar to our findings with iTBS application on the lower limb motor cortex, a meta-analysis on the effects of iTBS on the upper limb motor cortex revealed that the baseline MEP amplitude, age, muscle, and time of day may predict MEP changes after TBS (Corp et al., 2020). Additionally, the baseline MEP amplitude and time point predicted the MEP response following cTBS (Corp et al., 2020). Meanwhile, our study emphasized the significance of indicators reflecting intracortical excitability such as SICI and ICF, implying that inhibitory or facilitatory interneurons play important roles in TBS-induced plasticity in stimulated brain regions (Suppa et al., 2016; Li et al., 2019). Our initial research showed significant changes in SICI for both iTBS and cTBS applications to the lower limb motor cortex (Katagiri et al., 2020). Notably, a previous meta-analysis did not provide definitive conclusions on the long-term effects of TBS on SICI or ICF (Chung et al., 2016). The previous study by Di Lazzaro et al. (2001) suggests potential contributions from anatomical distinctions between the upper and lower limb motor cortices to diverse SICI circuit activation patterns. The conditioning TMS pulse of SICI appears to target distinct cortical layers in the upper and lower limb cortices due to I-wave composition-related differences (Di Lazzaro et al., 2001). These results suggest a potential difference in the origin of inhibition between the lower and upper limb motor cortices, and the SICI effects could potentially contribute to the observed discrepancy between our results and those of a previous study (Corp et al., 2020).

### 4.4 Clinical implications

According to the baseline neurophysiological characteristics of healthy participants before intervention, the Extra Trees model accurately classified responders to TBS. Interventions for post-stroke motor dysfunction in the upper and lower limbs using TBS are currently based on interhemispheric competition models (Chieffo et al., 2016; Huang et al., 2022; Vink et al., 2023). In the future, these models may improve post-stroke paralysis by customizing iTBS and cTBS to an individual’s responsiveness.

### 4.5 Limitations

Recognized contributors to plasticity after NIBS, including genetics, gender, and neural circuitry anatomy (Suppa et al., 2016; Huang et al., 2017), were not considered in our analysis, resulting in a notable limitation (Suppa et al., 2016; Huang et al., 2017). Future studies should address this limitation by including these important factors and more thoroughly examining the complex influences on neurophysiological outcomes particularly in the context of transcranial brain stimulation.

The study’s exclusive focus on healthy participants introduced a limitation concerning the generalizability of findings to individuals with specific neurological disorders such as lower limb paralysis. Additionally, the effects of NIBS may manifest differently in diverse populations (Huang et al., 2017; Ghasemian-Shirvan et al., 2020; Baharlouei et al., 2023). Therefore, caution should be observed when extrapolating our results to individuals with neurological disorders.

This study had a small sample size, which could limit the accuracy of the results. It only included 48 participants, indicating that this study was underpowered. However, we attempted to overcome this limitation by implementing analytical methods, such as random sampling and SMOTE. This study was a preliminary exploration that established the groundwork for future investigations targeting patients with central nervous system disorders. Subsequent studies will aim to validate the predictive factors identified in this study for effective outcome prediction.

### 4.6 Conclusion

Our results show that changes in cortical excitability after iTBS to the lower limb motor cortex may be accurately predicted using machine learning, whereas cTBS might be less precise. Predictive factors include corticospinal excitability for iTBS and intracortical excitability for cTBS. These findings suggest that the effects of TBS on the lower limb motor cortex are influenced by changes in cortical interneuron activity, which may vary depending on the TBS pattern. The findings also provide valuable insights into the diverse individual responses to TBS within the lower limb motor cortex while proposing solutions for interindividual variability. Finally, these results should contribute to the future application of TBS in the rehabilitation of patients with neurological conditions affecting the lower limbs.

## Data availability statement

The raw data supporting the conclusions of this article will be made available by the authors, without undue reservation.

## Ethics statement

The studies involving humans were approved by the ethics committee of the Faculty of Health Science of Juntendo University. The studies were conducted in accordance with the local legislation and institutional requirements. The participants provided their written informed consent to participate in this study.

## Author contributions

NK: Conceptualization, Investigation, Visualization, Writing – original draft, Writing – review & editing. TS: Conceptualization, Data curation, Formal Analysis, Methodology, Software, Visualization, Writing – original draft, Writing – review & editing. SS: Conceptualization, Funding acquisition, Writing – original draft, Writing – review & editing. ST: Software, Writing – original draft, Writing – review & editing. TY: Conceptualization, Funding acquisition, Project administration, Supervision, Writing – original draft, Writing – review & editing.

## Figures and Tables

**Table:** 

AMT	active motor threshold
AUC	area under the curve
cTBS	continuous theta burst stimulation
EEG	electroencephalography
ICF	intracortical facilitation
I-wave	indirection wave
ISI	interstimulus interval
iTBS	intermittent theta burst stimulation
MEP	motor evoked potential
MEP-CV	coefficient of variation of motor evoked potential
NIBS	noninvasive brain stimulation
RC	recruitment curve
RMT	resting motor threshold
ROC	receiver operating characteristic
SICI	short-interval intracortical inhibition
SMOTE	synthetic minority oversampling technique
tDCS	transcranial direct current stimulation
TBS	theta burst stimulation
TMS	transcranial magnetic stimulation
